# Promoting healthy aging in a digital world: leveraging technology for enhanced elderly care and wellbeing

**DOI:** 10.3389/fragi.2026.1687784

**Published:** 2026-02-04

**Authors:** Patrizio Armeni, Irem Polat, Leonardo Maria De Rossi, Lorenzo Diaferia, Sara De Padova, Athena Gatti, Severino Meregalli

**Affiliations:** 1 LIFT Lab, CERGAS GHNP Division, SDA Bocconi School of Management, Milano, Italy; 2 LIFT Lab, CERGAS GHNP Division, and DEVO Lab, Claudio Demattè Research Division, SDA Bocconi School of Management, Milano, Italy

**Keywords:** aging population, artificial intelligence, digital health technologies, digital inclusion, healthy aging, robotics, virtual reality, wearable sensors

## Abstract

The rising demands of an aging population underscore the need for digital health technologies (DHTs) in elderly care. Traditional services, which rely heavily on face-to-face, human-dependent interactions by medical and nursing staff, may struggle to meet these needs amid growing healthcare workforce shortages. This review examines the emerging role of DHTs—including wearable sensors, artificial intelligence (AI), robotics, and virtual reality (VR)—in supporting healthy aging. These technologies offer promising solutions to challenges such as chronic disease management, mobility impairments, cognitive decline, and social isolation. However, despite their potential, their adoption and integration into care systems remain limited due to a range of barriers. While we identify these barriers in detail, we also aim to open a broader discussion on why the urgency to act is particularly pronounced in Europe. The continent faces a sharp demographic shift, combined with uneven digital readiness across countries, under-resourced long-term care systems, and regional disparities in access to innovation. To fully harness the potential of DHTs in promoting healthy aging, this review outlines six strategic priorities: improving user-centered design; expanding real-world validation; promoting digital inclusion; addressing ethical and regulatory concerns; defining appropriate reimbursement and funding pathways; and innovating care delivery models. Advancing these areas will be critical to ensuring that DHTs contribute meaningfully to equitable and sustainable aging.

## 1 Introduction

The global demographic landscape is rapidly changing, driven by the rapid increase in the size and proportion of older persons. The share of the global population aged 65 years or above is projected to rise from 10 per cent in 2022 to 16 percent in 2050 (United Nations Department of Economic and Social AffairsPopulation Division, 2022). This demographic shift is challenging the capacity of many societies to meet the healthcare needs of an aging population, placing pressure on public health systems and social services. In response to aging populations, countries should adapt public programs to better support older adults, including by ensuring sustainable social security, universal healthcare, and long-term care systems (United Nations Department of Economic and Social Affairs Population Division, 2022).

To support healthy aging and ensure sustainable development, it is essential to prepare for the economic and social implications associated with an aging society. The World Health Organization (WHO) defined healthy aging as “the process of developing and maintaining the functional ability that enables wellbeing in older age” (Chen C. et al., 2023; Almutairi et al., 2022). This involves creating supportive environments and opportunities that allow people to live healthy, active lives as they age (World Health Organization, 2020). A key societal objective is to promote healthy aging by supporting older adults' autonomy and safety in their homes (Chen J. et al., 2023; Offermann et al., 2024). Digital health technologies (DHTs) present promising solutions to address the complex care needs of older adults in home environments, potentially enhancing traditional care practices and promoting independence among aging populations (Chen C. et al., 2023; Valokivi et al., 2023).

The rising demands of an aging population underscore the need for DHTs in elderly care. Traditional services, which rely heavily on face-to-face, human-dependent interactions by medical and nursing staff, may struggle to meet these needs (Lee C. H. et al., 2023). Elderly care also presents unique challenges, including managing multiple chronic conditions, cognitive decline, and end-of-life care (Abadir and Chellappa, 2024). Many individuals over 65 experience conditions marked by gradual cognitive and physical decline. Often, early symptoms progress unnoticed over several years and are only detected in advanced stages, when interventions are costly, and outcomes are less favorable. Early detection of these conditions is therefore essential, not only to prevent hospitalization and reduce the socio-economic costs of care but also to significantly enhance the quality of life for older adults (Almeida et al., 2019). Notably, a study by (Offermann et al., 2024) found high acceptance of technology among older adults and chronically ill participants, further supporting the case for integrating DHTs in elderly care.

The application of DHTs, particularly in the realms of wearable devices, artificial intelligence (AI), robotics and immersive technologies like virtual reality (VR) is rapidly advancing the field of elderly care. These technologies enable continuous remote monitoring and provide critical support in areas such as fall detection (Yacchirema D. et al., 2018), activity recognition (Debauche et al., 2019), and health status monitoring (Yacchirema D. C. et al., 2018; Zhang et al., 2020), all of which are essential for ensuring the safety and wellbeing of older adults living independently (Almutairi et al., 2022; Qian et al., 2021). These applications improve the quality of life for elderly individuals by detecting behaviors and providing proactive care (Almutairi et al., 2022).

The definitions of the terminology used in this paper are presented in Table 1.

**TABLE 1:** Definitions.

Term	Definition	Source
Digital health technologies	DHTs are tools that leverage computing platforms, software, connectivity, and sensors to support a broad range of health-related functions, from general wellness to direct medical applications. DHTs may serve as medical products themselves, be integrated into or used alongside medical products (such as drugs, biologics, or devices), or function as companion diagnostics. They are also commonly used in the development, monitoring, or study of medical interventions	US Food and Drug Administration (2020)
Wearable devices	“Wearables” is a term used for forms of technology that are worn on the body, such as smartwatches or adhesive patches containing sensors, and that perform a useful function for the wearer or a caregiver. Common examples include devices that track physical activity and sleep or provide physiological data about the wearer, such as heart rate and rhythm or blood glucose levels	Friend et al. (2023)
Artificial Intelligence	AI is a machine-based system that can, for a given set of human-defined objectives, make predictions, recommendations, or decisions influencing real or virtual environments. AI systems use machine- and human-based inputs to perceive real and virtual environments; abstract such perceptions into models through analysis in an automated manner; and use model inference to formulate options for information or action. AI includes machine learning, which is a set of techniques that can be used to train AI algorithms to improve performance of a task based on data	US Food and Drug Administration (2024a)
Robotics	Robotics refers to the science and practice of designing, manufacturing, and applying robots	International Organization for Standardization (2021)
Immersive technologies	Immersive technologies refer to a category of digital technologies that aim to immerse users in a simulated or augmented environment, typically including virtual reality, augmented reality (AR), and mixed reality (MR)	Li (2024)
Virtual Reality	VR is a virtual world immersive experience that may require a headset to completely replace a user’s surrounding view with a simulated, immersive, and interactive virtual environment	US Food and Drug Administration (2025a)
Natural-language processing (NLP)	NLP is an academic and technology-based research domain comprising a range of computational techniques for representation and automatic analysis of human languages	Chowdhary (2020)
Generative AI (GenAI)	GenAI is the class of AI models that emulate the structure and characteristics of input data in order to generate derived synthetic content. This can include images, videos, audio, text, and other digital content. This is usually done by approximating the statistical distribution of the input data. For example, in healthcare, generative AI can be used to generate annotations on synthetic medical data (e.g., image features, text labels) to help expand datasets for training algorithms	US Food and Drug Administration (2025b); Zohny, et al. (2023); Meskó and Topol (2023)
Chatbot	Chatbots, also known as conversational agents, interactive agents, virtual agents, virtual humans, or virtual assistants, are artificial intelligence programs designed to simulate human conversation via text or speech. In the context of healthcare, chatbots or healthbots are intended to provide personalized health and therapy information to patients, provide relevant products and services to patients, as well as suggest diagnoses and recommend treatments based on patient symptoms	Palanica et al. (2019)
Virtual assistants	The term “virtual assistant” refers to computer programs that are personified and created to communicate with one another and behave like humans. Virtual assistants are also increasingly being designed for various health applications, such as delivering cognitive behavior therapy for depression and anxiety, improving diet and physical activity, and conducting remote patient monitoring	Sahu et al. (2024)
Socially Assistive Robots (SARs)	Socially assistive robotics provide assistance to human users, and it specifies that the assistance is through social interaction. The robot’s goal is to create close and effective interaction with a human user for the purpose of giving assistance and achieving measurable progress in convalescence, rehabilitation, learningetc.	Feil-Seifer and Mataric (2005)
Artificial Emotional Intelligence (AEI)	Emotion AI is a subset of artificial intelligence (the broad term for machines replicating the way humans think) that measures, understands, simulates, and reacts to human emotions. It’s also known as affective computing, or artificial emotional intelligence	Somers (2019)
Game-based VR (exergames)	Exergames are considered as interactive video-games which require the player to produce physical body movements in order to complete set tasks or actions, in response to visual cue	Stanmore et al. (2017)
Dual-purpose aging	Dual-Purpose aging refers to the concept that the biological mechanisms underlying aging not only drive the aging process itself but also contribute to the development and progression of age-related diseases, such as cancer, fibrosis, metabolic, cardiovascular, and neurodegenerative conditions	Leung et al. (2024)

Although DHTs are progressing rapidly, their implementation for the elderly is still in its early stages (European Parliamentary Technology Assessment, 2019; Bertolazzi et al., 2024; Shen and Naeim, 2017). The current state of the field has yet to produce a robust paradigm that can be widely applied in real-world settings (Qian et al., 2021). Contributing to this developing field, this review aims at addressing the research questions below:

- What are the innovative technologies currently supporting elderly care and healthy aging?
- What specific challenges do aging individuals, caregivers, healthcare providers, and technology developers face in DHTs adoption?
- How prepared is the current landscape for the adoption and diffusion of DHTs?

The first research question explores DHTs applications in elderly care and healthy aging. Identifying these innovations will help mapping the current technological landscape and highlight emerging trends.

The second research question examines key adoption challenges from multiple stakeholder perspectives, including aging individuals, caregivers, healthcare providers, and technology developers. Understanding these challenges is crucial for assessing user needs, adoption barriers, and the design requirements necessary to enhance the usability and effectiveness of DHTs for older adults.

The final research question assesses the adoption and regulatory landscape of DHTs across Europe. As life expectancy increases and healthcare demands grow, older adults are becoming primary users of digital health innovations, including AI-based medical devices. European health systems must adapt to these shifts by fostering supportive policies and regulatory frameworks (Raja et al., 2021). Europe is facing fast aging populations and healthcare workforce shortages that require innovative digital solutions (Van Kolfschooten, 2023).

The rest of the paper is organized as follows. Section 2 presents the methodology. Section 3 addresses the first research question by examining the current state of DHT applications in elderly care and healthy aging. It provides an overview of the key technologies employed—such as artificial intelligence, robotics, wearable devices, and immersive technologies—highlighting their roles and emerging trends in supporting older adults. Section 4 responds to the third research question, analyzing regional differences in the adoption and regulation of these technologies across European countries. It explores how national policies, healthcare system structures, digital readiness, and regulatory frameworks shape the implementation of DHTs for aging populations.

Section 5 engages with the second research question by discussing the main challenges and open issues in implementing digital health solutions for elderly care. This includes perspectives from aging individuals, caregivers, healthcare providers, and technology developers, addressing critical concerns such as user acceptance, data privacy, ethical implications, usability, and disparities in access.

Section 6 offers a broader discussion, synthesizing insights across all three research questions and identifying key areas where further research, innovation, and policy action are needed to support the effective and equitable integration of DHTs in aging societies. Section 7 concludes the paper.

## 2 Methodology

This study follows a scoping review design. A scoping approach was chosen to map the breadth and characteristics of digital health technologies applied to elderly care, given the heterogeneity of technologies, study designs, and outcomes in this field. The aim was to map existing evidence, identify key themes, and highlight research and implementation gaps.

A total of 19,327 papers were retrieved from PubMed using a set of selection criteria, including specific keywords^1^ and a timeframe from 2014 to July 2024.

Initial text analysis of article titles was conducted to identify studies specifically related to older adults. We applied an automated keyword-based filter using ageing-related terms—“geriatr*,” “elder*,” “old*,” “aging,” “ageing,” and “senior” and retained only records whose titles contained at least one of these keywords. This step reduced the initial PubMed dataset from 19,327 to 2,925 articles and ensured that subsequent screening focused exclusively on studies relevant to older adults and their care environments.

Next, a text-labeling process categorized articles based on their titles, assigning labels such as artificial intelligence, robotics, wearables, VR and 5G—technology categories identified by the authors during a 2024 scouting phase on healthcare applications. This refinement reduced the dataset to 149 articles for title and abstract analysis, ultimately leading to the full-text inclusion of 68 articles. Additionally, 46 articles were added through the snowballing method, and 11 reports from grey literature were included.

## 3 The current state of DHTs applications on elderly people

There are high expectations on the potential of DHTs to improve the lives of older adults. DHTs can enhance communication, quality of life, and mobility for them and their caregivers. Current healthcare and social policies support “aging in place,” enabling older individuals to stay within their own homes and communities (Genge et al., 2023). In this setting, smart home technologies can help sustain and boost the abilities of those with physical or cognitive challenges (Pilotto et al., 2018). Moreover, mobile technologies enable continuous collection and processing of data for elderly’s health, activity, emotion, cognitive status in their living environments, allowing users to track trends and detect relevant changes (Stavrotheodoros et al., 2018; Durán-Vega et al., 2019; Mano et al., 2016). Home sensors can incorporate safety features, including bed and chair sensors, motion detectors, intruder alarms, and fall detectors (Pilotto et al., 2018; Koo and Vizer, 2019; Kulurkar et al., 2023; Hsieh et al., 2023). Sensors and robotic devices can assist older adults in improving their mobility and gait (Pilotto et al., 2018).

Based on existing literature, this review identifies four key digital technologies—wearable sensors, artificial intelligence, robotics and virtual reality—that play an increasingly important role in promoting healthy aging for older adults. This section explores these technologies, their applications, and the challenges associated with their implementation.

### 3.1 Wearable sensors

Traditional health assessments rely on in-clinic visits and annual tests, which are costly, inconvenient, and often lead to delayed diagnoses. This approach is particularly inadequate for continuous patient monitoring, especially given the increasing healthcare demands of the aging population (Chen J. et al., 2023). Wearable technologies can offer a promising solution by enabling early disease detection, personalized healthcare, and continuous health monitoring in non-clinical settings. Their widespread adoption, seamless connectivity, and advanced sensors allow for real-time data collection while reducing the burden on healthcare systems (Olmedo-Aguirre et al., 2022; Kourtis et al., 2019; Espay et al., 2016; Wang et al., 2017).

**FIGURE 1:**
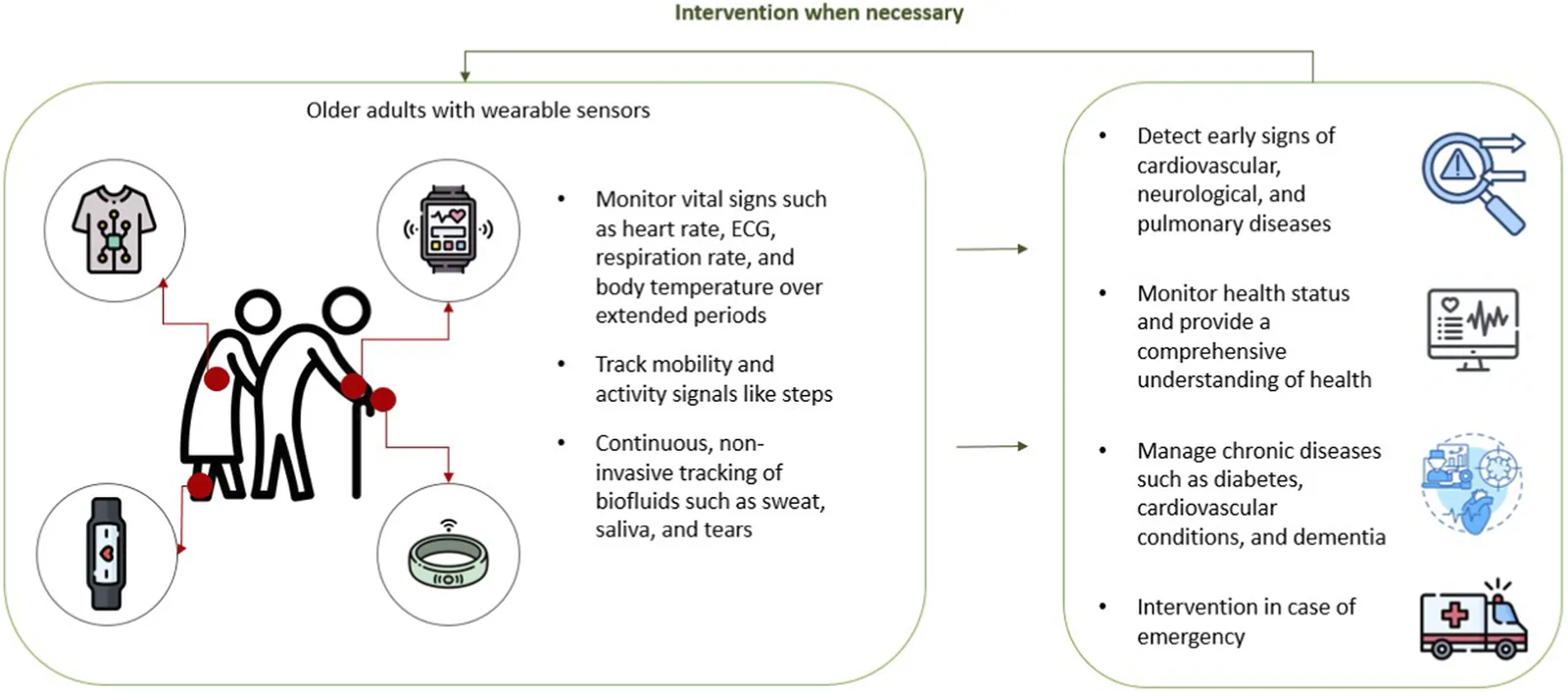
Wearable sensors in elderly care: Miniaturized and fabricated compact wearable technologies with multiple sensing modalities, which can be embedded in items such as smartwatches, smart rings, skin patches, and clothing.

The development and progress of wearable technologies have paved the way for the creation of effective devices for elderly care (Wang et al., 2017). Wearable sensors are now capable of gathering a broader range of physiological data, including vital signs, posture, movement, location, and sleep patterns (Shen and Naeim, 2017). By collecting extensive daily data, it becomes possible to establish personalized baseline assessments that can identify variations in an individual’s physiological parameters. Specifically, they track biological and physical factors of the metrics such as heart rate, blood pressure, respiration rate, electrocardiogram, and physical activity levels, offering valuable insights into health conditions and physiological changes in elderly populations (Chen C. et al., 2023; Shen and Naeim, 2017; Olmedo-Aguirre et al., 2022; Alharbi et al., 2019). This is crucial for elderly care, as illustrated in Figure 1. It is essential to quickly locate individuals during risky situations, enabling a prompt response from doctors and nurses to prevent further injury (Wang et al., 2017). For example, more accurate automated fall detection through wearables could enhance life-space and reduce the risk of falls (Godfrey, 2017).

(Kekade et al., 2018) found that a larger proportion of the population intended to use wearables to enhance physical and mental activities compared to other aspects of assisted living. Among the elderly, 55.11% (n = 70) aimed to improve physical activity, followed by 52.75% (n = 67) for mental activity, 37.7% (n = 48) for disease management, 35.4% (n = 45) for healthcare decision-making, and 31.49% (n = 40) for accessing health records. Similarly (Olmedo-Aguirre et al., 2022), reviewed in the literature the distribution of wearable devices among older adults, identifying the five most commonly mentioned types as watches (25%), bracelets (17%), patches (17%), intradermal sensors (13%), and portable sensors (8%).

These sensors now offer the flexibility to capture metrics through both active (prompted) and passive (unnoticed) measurements, broadening their range of applications and enhancing their utility in various health monitoring contexts. Kourtis et al. (2019) describe these two ways of data collection as below:

- Active data collection involves prompting the user to perform a task or input a metric, such as taking a digital cognitive test on a tablet to assess memory for Alzheimer’s Diseases or completing a voice test to check for vocal cord tremors linked to Parkinson’s. These measurements typically focus on specific metrics already associated with the disease.
- Passive data collection happens when data is gathered without the user’s awareness, such as a smartwatch tracking step symmetry and length, or a smart ring continuously monitoring heart rate variability. This ongoing interaction with mobile and wearable devices generates a detailed, high-frequency data set over time that can be analyzed for disease indicators. However, passive collection is limited to certain metrics, can be computationally expensive, and requires complex analysis. Passive data collection presents an enormous opportunity since they can be used by technology developers and Healthcare providers to provide a logical approach for developing methods for disease forecasting, detection and monitoring, and symptom management.

Considering adoption, older adults adopt new technology for its practical value rather than entertainment features (Moore et al., 2021). They view devices as tools and prioritize accuracy, reliability, and added value to their daily lives (Moore et al., 2021; Chen J. et al., 2023). The primary driver of adoption is the device’s purpose, such as fall detection or step counting, while factors like ease of use (e.g., battery life and touch screen menus), accuracy, and user experience influence long-term integration (Moore et al., 2021). While perceived cost negatively impacts elderly adults' intention to use wearable devices, aesthetic appeal has a positive effect. Additionally, personal innovativeness, physiological condition, and intention to use significantly influence actual usage behavior. Also, healthier older adults are less likely to adopt wearables, suggesting the need to identify target users and tailor features accordingly (Chen C. et al., 2023). When the drawbacks outweigh the benefits, adoption is unlikely (Moore et al., 2021).

Despite growing research on wearable devices in elderly healthcare, most studies focus on specific devices or applications, leaving a gap in understanding their comprehensive use. A key challenge is the accuracy and reliability of wearable devices in measuring health-related outcomes for older adults, as they are often designed with younger users in mind. Research remains inconclusive on their validity for assessing physical activity and health outcomes in elderly populations (Teixeira et al., 2021). Additionally, their continuous evolution through upgrades and redesigns highlights the need for ongoing research and reviews.

A major hurdle in wearable devices adoption among older adults is a lack of awareness regarding their benefits and usage (Kekade et al., 2018). However, the emergence of low-cost, high-fidelity wearable sensors with diverse clinical applications may drive increased adoption (Shen and Naeim, 2017). Establishing a standardized framework for wearable devices deployment and refining methods to calibrate, process, and utilize wearable data remain critical challenges (Alharbi et al., 2019; Godfrey, 2017).

### 3.2 Artificial intelligence

AI-driven solutions are increasingly used to address challenges in elderly care. Loveys et al. (2022) classify AI in this field into software-based systems operating in virtual environments (e.g., conversational agents, facial recognition systems) and hardware-based systems acting in physical environments (e.g., robots). Key AI techniques include machine learning, computer vision, pattern recognition, and natural language processing.

These AI-enabled interventions demonstrate potential in areas such as health prevention, screening, daily health assistance, and personalized treatments tailored to age and other patient-specific characteristics (Lee Y. H. et al., 2023; Shirini et al., 2023). Additionally, AI plays a role in predicting clinical outcomes such as mortality, hospital admission and readmission, delirium, survival rates, and fall prevention. It also aids in uncovering links between aging and diseases by identifying overlapping signaling pathways, common triggers, and synergistic effects, contributing to more effective and efficient research on aging and age-related conditions (Leung et al., 2024; Olender et al., 2023; Liu et al., 2023; O'Connor et al., 2022). Recent studies suggest that combining routine lab tests and diagnostic imaging with machine learning models can effectively predict cognitive and functional decline in the oldest-old population (Gomes et al., 2023; Abadir et al., 2024). Natural Language Processing (NLP) has also emerged as a valuable tool for enhancing the identification of aging-related syndromes, such as falls, dementia, and delirium, within electronic health records (Osman et al., 2024). Additionally, a growing body of empirical evidence highlights the potential of NLP to monitor and detect changes in speech and language due to neurocognitive aging and neurodegenerative processes. These subtle verbal modifications can be measured through NLP techniques and used as biomarkers for screening/diagnostic purposes in the aging population (Gagliardi, 2024; Zhavoronkov et al., 2019).

Beyond clinical applications, AI extends to enhancing the daily lives of older adults through a range of technologies, including robots (Abadir and Chellappa, 2024). These innovations not only assist in managing chronic conditions but also provide emotional support, companionship, and social engagement by addressing the psychological and social aspects of aging and contributing to a holistic approach to care for older adults. In summary, Table 2 provides an overview of the AI applications in elderly care.

**TABLE 2:** A summary of the AI applications in elderly care.

Applications	Description/Mechanism	Supporting evidence
Early detection and risk prediction	AI models leveraging routine labs, imaging, and speech/language biomarkers enable earlier identification of cognitive or functional decline	Gomes et al. (2023); Osman et al. (2024); Gagliardi (2024)
Personalized care and monitoring	ML algorithms support tailored interventions, medication optimization, and longitudinal tracking of multimorbidity	Lee C. H. et al. (2023); Shirini et al. (2023)
Enhancing independence and daily functioning	Vision, audio, and pattern-recognition systems support fall prediction, mobility assistance, and interpretation of behavioral trends	Qian et al. (2021); Almutairi et al. (2022)
Social and emotional support	Conversational agents and socially assistive robots use NLP and emotional-AI to address loneliness and cognitive engagement	Abadir and Chellappa (2024); Abdollahi et al. (2022)
Key challenges		
Challenges and concerns	Data bias, limited real-world validation, lack of representative older-adult datasets, and ethical concerns regarding privacy and manipulation	Karim et al. (2022); Leung et al. (2024)

AI in elderly care faces several critical challenges. A significant issue is the exclusion of older adults, particularly those with multiple chronic conditions, from clinical trials, which limits the applicability of machine learning models that primarily focus on single-disease diagnoses (Choudhury et al., 2020). Additionally, older adults often have reduced access to and familiarity with digital tools, increasing their risk of exclusion from AI research, despite evidence that tailored programs can enhance their technology adoption. This exclusion contributes to the development of biased models based on non-representative datasets, potentially leading to disparities in healthcare outcomes (Choudhury et al., 2020; Karim et al., 2022). The development of equitable AI models requires addressing socioeconomic, regional, racial, and ethnic disparities in training datasets. Without targeted funding and a proactive approach to designing fair algorithms, biased models could lead to negative clinical consequences (Karim et al., 2022). Moreover, AI applications in aging research are constrained by the lack of accurate longitudinal biological data. While synthetic virtual data could help mitigate this issue, this approach remains in its early stages and requires further validation, particularly for complex biological processes like aging (Leung et al., 2024).

Another major challenge is the limited real-world validation of AI applications in active assisted living. Most research has been conducted in controlled test environments, leaving a gap in understanding how these technologies perform in everyday settings with commercial devices. The absence of studies leveraging cloud technology further restricts large-scale deployment, as cloud-based solutions are essential for standardized data collection and management (Wang et al., 2024).

Addressing these limitations through better dataset representation and real-world testing will be crucial for advancing AI applications in elderly care.

#### 3.2.1 A perspective on generative AI (GenAI) as an emerging technology in elderly care

GenAI is becoming increasingly relevant in aging research (Leung et al., 2024). examined key advancements in the aging research over recent decades and identified GenAI as a major computational approach since 2023, particularly in the discovery of dual-purpose aging and disease targets. Although the literature on this topic remains limited, we highlight the potential of GenAI to enhance elderly care by addressing the unmet need for improved tools and services for older adults and caregivers. This underscores the importance of further research in this emerging field.

GenAI refers to a class of AI models that replicate the structure and characteristics of input data to generate new, synthetic content, such as images, videos, audio, text, and more (Executive Office of the President of the United States, 2023). Like predictive AI, GenAI learns patterns from data, but unlike predictive AI models, GenAI is designed to create new data resembling the original, rather than just identifying patterns for predictions. GenAI models analyze input data and produce contextually relevant outputs, which may not have been directly present in the training data (US Food and Drug Administration, 2024b).

##### 3.2.1.1 Currently deployable GenAI applications

GenAI can be implemented using current technology and have potential benefits for elderly care:

- Clinical documentation: Summarizing medical charts, lab results, and discharge notes to reduce caregiver workload (Jindal et al., 2024)
- Virtual assistant (human-like natural language-based form of interaction) using audio, video, physiological, and other sensors: Providing guidance on medication adherence, nutrition, physical activity, and social engagement (Chan et al., 2024; Fear and Gleber, 2023; Abadir et al., 2024). For instance, in this context, systems like ChatGPT are gaining attention for assisting Alzheimer’s patients and supporting caregivers (Abadir et al., 2024).

##### 3.2.1.2 Prospective/future GenAI applications

GenAI in elderly care are still emerging and largely speculative, requiring validation before widespread deployment. One potential application is using GenAI for social assistance, where it could be integrated with SARs or wearables to enhance social interaction and respond empathetically in real time. Another application could be automated immersive content creation, in which GenAI generates cognitive training or rehabilitation exercises specifically tailored to the needs of older adults.

### 3.3 Robotics

Over the past three decades, elderly-care robots have progressed from experimental research to real-world clinical applications, resulting in the development of various specialized robotic solutions. These include nursing robots for daily assistance, emotionally interactive companion robots, and intelligent robots designed for medical monitoring and physiological assessments (Zhao et al., 2023; Andtfolk et al., 2022). By integrating advanced robotics with the unique needs of elderly care, these technologies could help address challenges associated with demographic aging by improving safety, precision, and overall wellbeing for frail older adults (Zhao et al., 2023; Palmier et al., 2024). Most commercially available robots are designed for specific tasks, which aids older adults in home maintenance, allowing them to live independently for longer (Mois and Beer, 2020).

Robots are increasingly being developed not only for industrial use but also for everyday needs, including care settings. Human-interactive robots such as Socially Assistive Robots (SARs) which provide psychological enrichment by interacting with people and engaging their minds, are gaining popularity (Mois and Beer, 2020; Lee et al., 2022; Tanioka, 2019). By definition, SARs are a type of assistive technology designed to support human users through social interaction, leveraging this engagement to aid in areas such as convalescence, rehabilitation, and learning. Unlike other assistive robots, SARs prioritize meaningful exchanges with users to provide both social enrichment and functional support (Feil-Seifer and Mataric, 2005).

In elderly care settings, SARs are particularly valuable as they can support older adults by enhancing cognitive function, mental health and emotional wellbeing and can help combat loneliness (Gasteiger et al., 2021; Pu et al., 2019; Papadopoulos et al., 2022). To foster natural and effective interactions with older adults, SARs can be equipped with social capabilities and emotional intelligence, creating robots that are more empathetic, understanding, and responsive. Social robots with Artificial Emotional Intelligence (AEI), uses affective computing with artificial intelligence to evaluate or predict a person’s emotional state. These systems analyze data such as words, images, tone of voice, gestures, physiological signals, and facial expressions to assess feelings or emotions (Podoletz, 2023). For instance (Abdollahi et al., 2022), explored AEI integration in a SARs named Ryan, developed to engage seniors with depression and dementia. The study tested empathic and non-empathic versions of Ryan, where the empathic version could detect users' emotional states through multimodal recognition and respond appropriately, while the non-empathic version followed scripted dialogue. Results from a study with 10 senior care residents showed mood improvements with both versions, although the empathic Ryan was found more engaging and likable, as measured by interaction metrics and exit surveys.

The long-term success of SARs in elderly care depends on key design features and ethical considerations. Major concerns include risks of accidents, lack of reliability, loss of control, potential deception, social isolation, data privacy, and liability in case of safety issues (Palmier et al., 2024). Transparency is essential to prevent deceptive behaviors that may encourage short-term compliance but erode long-term trust (Robinson and Nejat, 2022). Additionally, cultural factors play a crucial role in shaping emotional models that ensure meaningful and relatable interactions. SARs must be adaptable to the diverse needs and preferences of older adults, requiring a deep understanding of the target population before implementation (Palmier et al., 2024; Koh et al., 2021; Pino et al., 2015). An example of a cross-cultural initiative is the EU-Japan Virtual Coach for Smart Ageing (e-VITA) project, which aims to develop a robotic care system tailored to healthy older adults' needs. The project involves adapting procedures to both European and Japanese users while ensuring compliance with ethical and legal regulations. Analyzing ethical concerns from users across different cultures within this framework could offer valuable insights (Palmier et al., 2024). Moreover, SARs must be designed to accommodate variations in user needs, as older adults living at home and those in care facilities have different expectations. Individuals with cognitive impairments may require additional support, while those with less technological experience often exhibit lower self-efficacy (Koh et al., 2021).

Research indicates that most older adults hold a moderately positive attitude toward SARs, expressing openness to their use, particularly for physical assistance (Luo et al., 2024). However, acceptance is influenced by various factors, including SARs’ physical appearance. Preferences regarding robot design vary widely, some older adults favor human-like features, while others prefer machine-like or hybrid appearances. Personalization options could enhance acceptance by allowing users to choose a design that aligns with their comfort and preferences (Mois and Beer, 2020; Vandemeulebroucke et al., 2021).

While the potential benefits of SARs in elderly care are widely recognized, objective evidence regarding their effectiveness remains limited (Lee et al., 2022; Koh et al., 2021). Many existing studies are conducted under controlled, short-term conditions rather than in real-world, long-term settings (Andtfolk et al., 2022; David et al., 2022), leaving limited evidence of the reliability and validity of SARs-based interventions (Van Patten et al., 2020; Loveys et al., 2022). For instance, there is insufficient validation of SARs-based interventions, particularly for individuals with dementia (Koh et al., 2021). Key research limitations include small sample sizes, lack of external validation, poor outcome reporting, and difficulties in collecting follow-up data from older adults in long-term care settings due to illness, fatigue, or mortality (Loveys et al., 2022). Additionally, comparing different studies without considering variations in robot design and functionality may lead to misleading conclusions, underscoring the need for further research on how appearance influences perceptions (Andtfolk et al., 2022). Many SARs remain in prototype stages, lacking the robustness of commercially available technologies such as smartphones (Pu et al., 2019). Technical challenges, including slow response times and execution errors, further hinder their practical implementation (Andtfolk et al., 2022).

To improve SARs effectiveness and acceptance, future research should employ validated outcome measures, conduct large-scale randomized controlled trials, and focus on long-term studies (Andtfolk et al., 2022; Loveys et al., 2022). Clarifying the specific roles SARs are intended to fulfill and demonstrating their ability to address real-world challenges will help transition them from novelty to practical functionality in elderly care (Abdi et al., 2018). A potential strategy to increase acceptance is the early introduction of robots into older adults’ lives, as age-related declines in physical, perceptual, and cognitive abilities may make later adoption more difficult (Mois and Beer, 2020).

### 3.4 Virtual reality

VR is emerging as a valuable tool in supporting older adults in managing age-related challenges, including musculoskeletal decline and chronic conditions that impact physical, cognitive, and psychosocial wellbeing (Dermody et al., 2020). Game-based VR (exergames) enhances engagement through interactive training, while non-game-based VR replicates real-world conditions and incorporates sensors for feedback, making exercise programs adaptable to individual needs (Baragash et al., 2022). Despite challenges such as sample selection criteria, limited testing of immersive technologies, and study biases, VR systems demonstrate good usability and acceptance among older adults (Tuena et al., 2020).

VR-based interventions have shown promising benefits in enhancing cognitive function, quality of life, activities of daily living, and emotional wellbeing in older adults (Wen et al., 2024; Yan et al., 2022). VR-based cognitive rehabilitation therapies are at least as effective as traditional cognitive rehabilitation, with additional advantages in motivation and engagement (Tortora et al., 2024). VR has also shown a positive effect on cognitive flexibility, global cognitive function, attention, and short-term memory in older adults with Mild Cognitive Impairment (Yu et al., 2023; Sakaki et al., 2021; Zhu et al., 2021).

One of the key advantages of VR is its adaptability, allowing for personalized rehabilitation through real-time adjustments in task difficulty (Wen et al., 2024). The flexibility of VR also enables safe training for potentially hazardous situations and targeted cognitive exercises (Bauer and Andringa, 2020). Additionally, VR serves as a valuable screening and assessment tool, demonstrating validity comparable to traditional paper-based cognitive tests (Skurla et al., 2022). The high-resolution data collected in VR environments can help detect subtle cognitive changes over time, which is particularly relevant for aging and dementia care (Bauer and Andringa, 2020). However, further refinements are needed to improve diagnostic specificity (Skurla et al., 2022).

Another area where VR exercise training has proven beneficial is in enhancing gait quality and resistance in healthy older adults, potentially helping to prevent declines in functional mobility (Corregidor-Sánchez et al., 2021). For older adults with frailty, regular VR exercise training provides a convenient and accessible method to improve walking speed and balance. Studies show that VR exercise training leads to significant improvements in both walking speed and balance (Lee Y. H. et al., 2023; Liu et al., 2022). A key limitation of current studies is the use of commercial exergames, which aren't tailored for older residents in long-term care facilities or specific intervention goals. While VR-based interventions may improve physical health in these settings, the evidence is still uncertain. Future research should focus on both the effectiveness and feasibility of implementing VR interventions in long-term care facilities (Li et al., 2022).

Despite these benefits, several challenges remain. Limited literature necessitates further high-quality studies to validate the effectiveness of VR interventions for older adults. While caregivers report positive perceptions, they must still consider factors such as accessibility, user-friendliness, cognitive adaptability, and technical support when selecting VR technology, ensuring the appropriate level of immersivity and device type for individual needs (Tortora et al., 2024; Flynn et al., 2022). Additionally, immersive VR environments can cause cybersickness, fatigue, and disorientation, impacting usability, while goal-oriented VR tasks may be too complex for individuals with dementia, potentially reducing their effectiveness (Tortora et al., 2024; Zhu et al., 2021; Bauer and Andringa, 2020; Flynn et al., 2022). Thus, issues like frailty, usability, and acceptability must be addressed before making stronger recommendations on VR’s effectiveness for older adults (Dermody et al., 2020).

To maximize the potential of VR in elderly care, future research should focus on standardizing VR environments for better cross-study comparisons. Finally, many existing studies originate from high-income countries, limiting the generalizability of findings to other populations (Skurla et al., 2022).

## 4 Regional differences in technology adoption for elderly care in Europe

As might be expected given the substantial differences among European countries in welfare organization, territorial structuring, and digitalization levels among government agencies and citizens, there are also variations in their approaches to technology adoption in the elderly care (Valokivi et al., 2023). There are several studies underlining the differences.

Merkel and Hess (2020) explored the use of digital health services among the elderly across Europe, revealing the highest adoption rates in Scandinavian countries and Estonia, and the lowest in Malta, Cyprus, and Germany. While internet-based health services offer significant advantages, the majority of older adults do not use them. The likelihood of using these services is influenced by socioeconomic factors and living environment, with those in rural areas or with lower socioeconomic status being more likely to be excluded, despite the potential benefits of remote healthcare. This digital divide can exacerbate social inequality, as access to technology becomes a key factor in health knowledge, communication, decision-making, and service utilization.

Valokivi et al. (2023) analyzed policy differences in eHealth approaches on digital ageing among Italy, Finland, and Sweden from 2009 to 2019. In comparing these three countries, notable differences emerge in their approaches to eHealth. Italian policy documents generally lack a critical perspective on the impact of technological changes on healthcare, older adults, and the relationships between users and healthcare professionals. They adopt a predominantly optimistic stance, focusing on the benefits of digitalization for society, the healthcare system, and citizens. In contrast, Finnish and Swedish documents show a more nuanced approach, addressing ethical considerations, potential risks, and the importance of user involvement—including input from older adults and healthcare professionals—early in the digitalization process. Nordic policies thus emphasize a more balanced perspective, while Italy leans towards a techno-positive view with limited attention to potential complexities or risks.

Möller et al. (2024) introduced the e-VITA virtual coach, a Technological Coaching System designed to empower older adults through a socio-technological support system in Europe and Japan. The study aims to understand the digital behavior and profiles of older adults, distinguishing between the internet users and non-users. Data from the 2018 European Social Survey for Italy, France, and Germany, alongside Japanese data from the 9th International Comparative Survey, revealed that over half of adults aged 65 and above in Germany (56.96%) and France (59.9%) use the Internet. However, only 39.14% of Italian seniors in this age group are internet users. Further studies indicate that in Italy, older adults aged 65–74, especially those with lower socioeconomic status, represent a minority among digital users, highlighting a digital divide. Internet use is linked to better socioeconomic conditions, physical activity, larger social networks, and greater personal satisfaction.

Akenine et al. (2020) explored the attitudes of older adults at higher risk of cardiovascular disease and dementia towards eHealth self-management prevention programs, focusing on facilitators and barriers. The study included 44 participants aged 65+ from Finland, France, and the Netherlands. It found that Finnish participants were more independent and took greater responsibility for their health decisions, whereas Dutch and French participants tended to rely more on their healthcare providers. These differences were linked to variations in the healthcare systems of each country, with Finland focusing more on patient autonomy compared to the Netherlands and France, where doctor-patient relationships are more stable. The study concluded that creating a secure and trustworthy online environment could boost participation in prevention programs and reduce the stigma surrounding dementia. It also highlighted the importance of tailoring prevention initiatives to the local healthcare culture and context when planning international programs.

The readiness for adoption and the actual adoption of DHTs for elderly care varies widely across Europe. Differences in healthcare systems and socio-economic factors influence the use of these technologies, highlighting a digital divide. To foster greater engagement, tailored and culturally adapted initiatives are needed to ensure that older adults across Europe can fully benefit from digital health services, mitigating the risk of exacerbating existing social inequalities.

## 5 Challenges and open issues

Advancements in DHTs offer promising potential to improve care for older adults, though their impact is still emerging. Several factors hinder wider adoption, and this section highlights key challenges.

### 5.1 Reluctance among older adults to adopt DHTs

Older adults often show reluctance to adopt DHTs. Factors such as privacy risks, performance concerns, legal issues, and trust have been found to significantly influence their intention to use mobile health apps, wearable sensors, or activity recognition tools for daily life behavior analysis (Klaver et al., 2021). The topic of privacy, especially in relation to health monitoring has been widely discussed in the literature (Felber et al., 2023). Privacy concerns in elderly care vary among users, with some older persons less concerned about privacy than other factors like cost and usability, and willing to trade privacy for safety and the ability to stay at home. Both older individuals and caregivers value control over technology, data collection, and monitoring locations. Issues around data theft, dissemination, and the impact of technology on behavior and relationships are also significant. Additionally, debates arise about whether more intrusive privacy measures should be allowed for those with cognitive impairment or if privacy should be equally protected for all users.

Certain age-related characteristics, including hearing loss, limited dexterity, and low vision, can further impact their comfort with new technologies. Studies have shown that common barriers to adoption include discomfort with wearing smart home sensors, disruptions to daily routines, and usability challenges, such as difficulty controlling and interpreting sensor functions (Alharbi et al., 2019; Moore et al., 2021; Jo et al., 2021; Isabet et al., 2021). A real-world example addressing these challenges comes from South Korea, where an AI speaker solution was implemented to support health-vulnerable populations. In 2021, an Android-based AI speaker was upgraded to include a display, allowing the integration of Today’s Health®, an app designed for senior citizens. This upgrade enabled older adults, particularly those from vulnerable groups, to participate in the project without technological limitations (Kim et al., 2024).

Additionally, many mobile health apps suffer from design flaws (Qian et al., 2021; Li et al., 2021). In the case of robotic devices, unfamiliar or humanoid appearances may create fear, leading to reduced interaction among older users (Tian et al., 2024). To enhance adoption, selecting sensors and devices that align with users' needs and environments is essential (Felber et al., 2023). Clear privacy policies and guidelines can reassure older users about data security, enhance awareness of DHT benefits, and support a better understanding, promoting potential adoption of these technologies.

Another reason is the familiarity with the technology. For instance (Ho et al., 2023), found significant positive correlation between familiarity with AI applications and perceptions of emotional AI in both private and public medical settings. This raises the implication that senior patients, who portrait more negativity toward emotional AI applications, may gradually come to accept them given enough interaction with the technologies. Education and media can act as a platform for increasing familiarity with emotional AI application, thus producing a positive impact on emotional AI perception in healthcare (Ho et al., 2023).

Finally achieving a balance between human and artificial care in elderly support is crucial. This theme holds particular significance, as caregiving for older individuals has traditionally been a deeply human-centric activity. The debate on human versus artificial caregiving revolves around four key concerns (Felber et al., 2023). First, there is a general fear that robots will replace human caregivers, leading to job losses, a lack of real interpersonal contact, and increased social isolation. Second, the importance of human caregiving is emphasized, with many arguing that human touch, relationships, and attention to health decline during in-person visits cannot be replaced by technology. Third, there are positive reactions to technology performing caregiving tasks, with some participants even forming attachments to robots or finding them beneficial for socialization. Finally, the idea of collaboration between humans and machines in caregiving is proposed, with some concerns about potential harms, such as caregivers withdrawing or reinforcing oppressive care dynamics. Opinions on whether caregiving robots should have life-like features, such as voices or emotional expressions, vary, reflecting current technological limitations. Future advancements in technology design and caregiving will need careful consideration from stakeholders to decide how much human touch and care should be maintained.

### 5.2 Limited technological experience among caregivers

Another issue concerns caregivers, who often have limited experience or knowledge of these technologies (Dino et al., 2022). According to (Tuisku et al., 2023) although nursing staff generally have limited experience with care robots, there is significant openness to training and assisting others in using this technology, underscoring the importance of targeted training programs for successful adoption. While care robots are increasingly integrated into welfare services across Europe, assistant nurses in countries like Finland, Germany, and Sweden show a limited background in training others on this technology. Only 11.3% of surveyed nurses had previously provided orientation for care robot use, revealing a potential knowledge gap. Still, more than half of respondents expressed a willingness to engage with the technology and support others in its use.

### 5.3 Knowledge gaps in DHTs among elderly care networks

One of the key concerns is the limited attention given to how increased digitalization in healthcare affects the support networks around older adults. It is essential to consider informal caregivers, family members, and friends in the rollout of DHTs and the digitalization of elderly care (Valokivi et al., 2023; Almeida et al., 2019; Isabet et al., 2021). For instance, implementation typically involves two essential but time-consuming steps: first, training older adults through presentations and demonstrations, and second, training professionals and family members to effectively use these tools (Isabet et al., 2021). Additionally, technological competency and literacy among both older adults and their care partners are crucial factors throughout the adoption and post-adoption phases (Mois and Beer, 2020).

Community engagement also plays a significant role (Ho et al., 2023). found that community activity and access to mobile internet have been shown to positively influence attitudes toward emotional AI applications. However, concerns about network approval also play a role in adoption. Older adults worry that family members or the public may disapprove of their use of technology, fearing unwanted attention. Moreover, family members often expect to be involved in the decision-making process regarding the adoption of new technologies (Tian et al., 2024).

To enhance acceptance and trust, researchers recommend organizing workshops in community centers to familiarize older adults and their caregivers with these technologies, fostering confidence and reducing concerns about adoption.

### 5.4 Elderly’s limited involvement in technology research and design

Older adults often feel uncomfortable or distrustful toward AI and digital technologies, largely due to their limited involvement in tech research and design processes, which has resulted in a lack of tailored solutions (Abadir et al., 2024). During the research and design phases, older adults remain underrepresented in clinical studies despite their burgeoning population. There are several major barriers to their recruitment in aging-related research, such as complex health issues, social and cultural factors, and challenges with obtaining informed consent, especially in cases involving cognitive impairments like dementia (Abadir et al., 2024; Wangmo et al., 2019).

Furthermore, recruiting older adults with chronic conditions is complicated by practical challenges, such as reduced vision, cognitive changes, and sometimes difficulty with consent. Limited tech interest or unintuitive interfaces can also discourage older adults, caregivers, or healthcare staff from engaging with certain digital features (Mody et al., 2008). In settings like nursing homes, residents are often safeguarded by staff and families who may mistrust research, fearing exploitation or the perception of residents as “being experimented on.” Staff may also worry about added workload and feel wary of external observation of their care routines. These concerns create significant barriers to involving older adults in tech research and development (Mody et al., 2008).

However, the limited involvement or exclusion of older adults in technology development presents a key challenge: the underrepresentation of elderly-specific data, giving rise to “ageism as a bias” in technology design for this demographic. To counteract digital ageism, future research and policy initiatives must actively integrate inclusivity into their agendas (Chu et al., 2022). An inclusive co-design approach, involving both caregivers and older adults, is essential to ensure that technological solutions are genuinely aligned with their real-world needs and experiences (Deusdad, 2024).

### 5.5 Limited evidence from real-world settings

Evidence for the effectiveness of aging-related technology still needs to be established (Kim et al., 2021). Although many applications aim to support elderly care, their evaluation in real-world contexts remains limited. Most studies occur in controlled settings, like labs, which differ from real-life environments. For instance, fall detection data often comes from stunt actors simulating falls in labs, which may not accurately reflect real-world falls (Qian et al., 2021). Similarly, digital linguistic biomarkers created through NLP techniques can aid in screening or diagnosis for elderly populations, yet few studies have effectively integrated these tools into clinical practice (Gagliardi, 2024). In the field of robotics, common study limitations include a lack of detailed methodological reporting, the absence of randomized controlled trials, and inadequate real-world testing (Shishehgar et al., 2019). Setting realistic research goals at a population level and supporting technology-based interventions with continuous data collection and sharing from real-world settings are needed (Qian et al., 2021; Kim et al., 2021).

### 5.6 Cost and unfair access to the technology

At a societal level, the cost of technology can be prohibitive for many end-users, particularly those with lower socioeconomic status (Wangmo et al., 2019). Limited internet access and inadequate household space for technological devices further exacerbate issues of equitable access, particularly in rural or remote areas (Bertolazzi et al., 2024). For instance, in the case of robots, the high cost and limited availability not only hinder large-scale research but also contribute to the underrepresentation of low- and middle-income countries in studies (Loveys et al., 2022).

Educational level also plays a critical role in technology adoption. Well-educated older adults are more likely to adopt and use digital technologies, while lower education levels can hinder acceptance and use. Addressing these disparities through targeted policy solutions is essential to ensure that clinically beneficial technologies reach those in need (Bertolazzi et al., 2024). Addressing these access disparities with targeted policy solutions is essential to ensure that clinically beneficial technologies reach those in need (Wangmo et al., 2019).

## 6 Discussion

The findings of this review highlight the growing role of DHTs in promoting healthy aging, particularly through wearable sensors, AI, robotics, and VR. These innovations offer promising solutions for addressing the unique challenges of aging populations, including chronic disease management, mobility limitations, cognitive decline, and social isolation. However, as synthesized in Table 3, the maturity and real-world integration of these technologies vary considerably, reflecting persistent gaps between technological development and sustainable adoption in elderly care.

**TABLE 3:** Maturity levels and adoption barriers of digital health technologies for healthy aging.

Technology	Applications in healthy aging	Maturity level	Key limitations and adoption barriers
Wearable sensors	Activity and mobility monitoring, fall detection, sleep tracking, vital sign monitoring	Real-world	Privacy concerns related to continuous monitoring; usability issues due to dexterity, vision, or comfort with wearing devices; data accuracy for frail older adults; interoperability and data governance challenges
Artificial Intelligence	Risk prediction, screening, decision support, NLP-based monitoring	Pilot/Real-world	Limited real-world validation; biased and non-representative datasets due to under inclusion of older adults; lack of trust and transparency; low familiarity with AI; privacy and legal concerns; limited caregiver experience
Robotics (SARs)	Physical assistance, companionship, cognitive and emotional support	Pilot	High costs and unequal access; ethical concerns (loss of human care, deception, autonomy); fear or discomfort due to appearance; limited caregiver training; technical reliability issues; lack of long-term real-world evidence
Virtual reality	Cognitive rehabilitation, physical training, assessment	Conceptual/Pilot	Usability and accessibility challenges; cybersickness and fatigue; cognitive burden for frail or cognitively impaired users; lack of standardization; limited feasibility in long-term care settings; scarce real-world evidence
GenAI	Conversational support, activity interpretation	Conceptual	High ethical and safety risks (manipulation, scams); lack of empirical validation; limited familiarity among older adults; trust and privacy concerns; minimal real-world deployment

Claims regarding user acceptance, ethical concerns, and system-level barriers are now supported by findings from systematic reviews (e.g., Moore et al., 2021; Loveys et al., 2022), meta-analyses (e.g., Lee C. H. et al., 2023; Corregidor-Sánchez et al., 2021), and empirical implementation reports (e.g., Valokivi et al., 2023; Merkel and Hess, 2020).

Building on this synthesis, four interrelated themes emerge that explain why technological maturity has not yet translated into routine, large-scale use in elderly care.

First, adoption challenges coupled with a non-mature societal readiness. One of the major challenges identified is the reluctance of older adults to adopt new technologies. While studies indicate a generally positive attitude toward DHTs when their utility is clear, factors such as unfamiliarity, usability concerns, cost, privacy risks and the loss of human interaction limit their adoption. Moreover, many elderly individuals prioritize ease of use and reliability, suggesting that more user-centered designs are needed to enhance acceptance. Socioeconomic and regional disparities further exacerbate these adoption challenges. For instance, internet access and digital literacy vary significantly across European countries, influencing older adults’ ability to engage with digital health services. Countries such as Sweden and Finland exhibit higher adoption rates of eHealth solutions, while others, including Italy and Germany, show lower levels of digital engagement among older populations. These differences underscore the need for tailored policy interventions that consider local healthcare infrastructures and digital literacy levels.

Second, unclear evidence of effectiveness brings consequences to the real-world implementation of these technologies. Indeed, despite the potential of AI and robotics in elderly care, real-world validation of these technologies remains limited. Most studies have been conducted in controlled environments, raising concerns about their reliability and scalability. For instance, fall detection algorithms trained on simulated data may not perform as effectively in real-life scenarios. Similarly, SARs have demonstrated positive effects on cognitive and emotional wellbeing, but their long-term impact and cost-effectiveness require further investigation. More large-scale, real-world trials are needed to assess the feasibility and outcomes of these technologies in diverse care settings. The integration of AI in elderly care also presents ethical concerns, particularly regarding biased algorithms and the exclusion of older adults from machine learning model training. AI models often rely on datasets that may not represent the complexities of multimorbidity in aging populations, leading to disparities in clinical outcomes. Ensuring diverse and representative data, alongside rigorous ethical oversight, is crucial for developing equitable AI-driven healthcare solutions.

Third, the role of caregivers and healthcare providers. Another key finding of this review is the critical role of caregivers and healthcare professionals in mediating, facilitating or hindering the adoption of DHTs. Many caregivers, including both family members and professional staff, lack sufficient training in using AI-driven and robotic solutions, limiting their ability to support older adults in technology adoption. They may, indeed, be one driver of a missed adoption. Targeted education programs and workshops could help bridge this gap, ensuring that caregivers can effectively integrate these tools into daily care routines. Healthcare providers also face challenges in integrating DHTs into existing care models. While wearable sensors and remote monitoring tools can improve early disease detection and personalized treatment, interoperability with healthcare systems remains a barrier. Standardized frameworks for data sharing, privacy protection, and regulatory compliance are necessary to ensure the seamless integration of digital solutions in elderly care.

Fourth, inconsistent and often lacking financial support and integration into existing service models. A critical challenge in the widespread adoption of DHTs is the issue of reimbursement and funding within public or insurance-based healthcare systems. Many of these technologies, including AI-driven diagnostics, robotic caregivers, and smart home monitoring systems, require substantial investment, yet reimbursement models for such innovations remain underdeveloped. In many countries, existing public healthcare systems and insurance policies do not adequately cover digital health interventions for elderly care, leaving gaps in affordability and access. For DHTs to be effectively integrated into elderly care, healthcare systems must adapt their reimbursement policies. This could include new financing mechanisms such as value-based reimbursement models, in which healthcare providers are compensated based on patient outcomes rather than the volume of services provided. Additionally, there is a need for clearer regulatory frameworks that define which technologies qualify for reimbursement and how their clinical benefits are assessed. Another key issue is the integration of DHTs into existing service models. Traditional elderly care services are often structured around in-person interactions and institutional care, making the integration of remote monitoring, AI-based diagnostics, and assistive robotics challenging. To accommodate these emerging technologies, new service models must be developed that incorporate hybrid care approaches, blending in-person and digital interventions. These models should be designed with a focus on patient-centered care, ensuring that older adults receive personalized and effective support that aligns with their needs and preferences.

Overall, these findings indicate that overcoming the barriers to adopting digital health technologies in elderly care requires coordinated efforts across research, practice, and policy. Building on the challenges identified in this review, the following sections outline key priorities for researchers, healthcare providers, and policymakers to support effective and sustainable implementation.

Researchers should first focus on significantly strengthening real-world validation of digital health technologies. This requires conducting large, longitudinal, and methodologically rigorous studies that assess usability, safety, and clinical outcomes in naturalistic environments such as homes, assisted living facilities, and outpatient clinics. Evidence generated from real-world use is essential for evaluating the true effectiveness and scalability of artificial intelligence, robotics, wearable sensors, and virtual reality in elderly care. A second priority for the research community involves advancing user-centered and co-design approaches. Engaging older adults, caregivers, and healthcare professionals throughout the development process ensures that technological solutions are intuitive, ergonomically appropriate, and aligned with the physical, cognitive, and emotional needs of the people who will ultimately use them.

Practitioners and healthcare providers play a critical role in ensuring that digital health technologies deliver value in day-to-day care. Their most pressing priority is the innovation and adaptation of service delivery models so that digital tools are seamlessly integrated into clinical workflows. This includes combining telemedicine with in-person care, incorporating remote monitoring data into clinical decision-making, and using robotics to support daily activities within long-term care settings. A complementary recommendation concerns the development of workforce capacity. Providers must receive adequate training in the use of digital tools, the interpretation of sensor-based or AI-generated data, and the ethical considerations involved in technology-mediated care, so that they can confidently and effectively support older adults in adopting these innovations.

Policymakers have an essential role in shaping the conditions under which digital health technologies are deployed. Their highest priority is the promotion of digital inclusion and equity. This involves investing in digital literacy initiatives, community-based training programs, and infrastructure improvements that ensure older adults from all regions and socioeconomic backgrounds can benefit from technological advancements. A further recommendation for policymakers concerns the creation of clear ethical, regulatory, and data governance frameworks. These frameworks should address algorithmic fairness, privacy protection, data security, and transparency in consent processes, thereby ensuring that digital health solutions are implemented responsibly and without reinforcing existing inequities. Finally, policymakers should work to establish sustainable reimbursement and financing mechanisms that reflect the value of digital interventions. Supportive funding models for remote monitoring, AI-assisted diagnostics, and assistive robotics, along with grants or subsidies for individuals and institutions, can substantially improve adoption.

## 7 Conclusion

Based on the extensive literature review conducted, we conclude that while DHTs offer transformative potential in elderly care, their successful implementation demands a comprehensive, multidisciplinary approach that encompasses technological innovation, healthcare system adaptation, and supportive policy frameworks. To fully harness this potential, several critical areas require further attention. First, improving user-centered design is essential to ensure accessibility, ease of use, and personalization, with co-design approaches that engage older adults directly. Second, real-world validation through large-scale, longitudinal studies and multi-center collaborations is needed to demonstrate the efficacy and scalability of technologies such as wearable sensors, AI, robotics, and VR in diverse elderly care settings. Third, digital inclusion strategies must be strengthened by investing in digital literacy, training programs, and internet access—particularly in underserved regions. Fourth, ethical and regulatory considerations must be prioritized, including clear frameworks for data privacy, informed consent, and algorithmic fairness to prevent biases and health disparities. Fifth, developing appropriate reimbursement pathways and financial incentives will be crucial in encouraging adoption by healthcare providers and reducing costs for older adults and institutions alike. Finally, innovative service models should be promoted that integrate digital and physical care—such as hybrid telemedicine approaches, connected monitoring systems, and assistive robotics—while equipping healthcare professionals with the necessary skills to manage these new tools effectively.

By addressing these interconnected challenges and fostering a more inclusive and ethically grounded digital landscape, public health policies can better align with technological advancements. This will enable societies to support aging populations in maintaining independence, improving wellbeing, and enhancing their quality of life.

## Footnotes

1 Search query (PubMed): (Healthcare OR Care OR Medicine OR Medical) AND (Ageing OR Elder OR Elderly OR Older adult OR Older adults) AND (technology OR digital OR artificial intelligence or AI or virtual reality or VR or IoT or internet of things OR mhealth or ehealth or e-health) Filters: Editorial, Review, Systematic Review, from 2014–2024.

## Supplementary material

SUPPLEMENTARY TABLE S1 Code for text-labeling process used to categorize articles into technology domains.

SUPPLEMENTARY TABLE S2 Key characteristics of the included studies.
