# Correction: Promoting healthy aging in a digital world: leveraging technology for enhanced elderly care and wellbeing

**DOI:** 10.3389/fragi.2026.1988532

**Published:** 2026-09-30

**Authors:** Patrizio Armeni, Irem Polat, Leonardo Maria De Rossi, Lorenzo Diaferia, Sara De Padova, Athena Gatti, Severino Meregalli

**Affiliations:** 1 LIFT Lab, CERGAS GHNP Division, SDA Bocconi School of Management, Milano, Italy; 2 LIFT Lab, CERGAS GHNP Division, and DEVO Lab, Claudio Demattè Research Division, SDA Bocconi School of Management, Milano, Italy

**Keywords:** aging population, artificial intelligence, digital health technologies, digital inclusion, healthy aging, robotics, virtual reality, wearable sensors

Affiliation “LIFT Lab, CERGAS GHNP Division, SDA Bocconi School of Management”, was omitted from the paper. This affiliation has now been added for author(s) Athena Gatti.

Affiliation “International School of Bologna” was erroneously included in the paper. This affiliation has now been removed for author(s) Athena Gatti.

The original version of this article has been updated.

