# Supplementary material for: Promoting healthy aging in a digital world: leveraging technology for enhanced elderly care and wellbeing
Source: Front Aging. 2026 Feb 4;7:1687784. doi: 10.3389/fragi.2026.1687784 (PMC12913556; doi:10.3389/fragi.2026.1687784)
Supplement: Supplementary file 2 [file Table1.docx]

**Supplementary File: Code for Text-Labeling Process Used to Categorize Articles into Technology Domains (in Python)**

**Code:**

import pandas as pd

import numpy as np

df = pd.read_excel('Pubmed article search results.xlsx')

data = df.copy()

data['Title']= data['Title'].astype(str)

words_ai = ['Artificial intelligence', ' AI,', ' AI-', ' AI ', ' AI.', 'Machine Learning', 'Predictive Analytics', 'automl', 'Natural Language Processing', 'NLP', 'semantic search', 'sentiment analysis', 'natural language generation', 'natural language understanding', 'speech-to-text','speech to text' ,'text-to-speech','text to speech', 'speech recognition', 'Text Analytics', 'chatbot', 'userbot'] words_iot = ['Internet of Things', ' IOT ', ',IOT '] words_5G = ['5G'] words_vr_ar = ['Virtual Reality' , ' VR ', ' VR,', ', VR ', 'Augmented Reality', ' AR ', ' AR,', ', AR ', 'VR/AR'] words_robotics = ['Robotic', 'robot'] words_digitalliteracy = ['literacy'] words_regulation_policy = ['regulation', 'policy', 'initiative', 'regulat']

def ai(x): for i in words_ai: if i.lower() in x.lower(): return 'Artificial Intelligence' else: return np.nan

def iot(x): for i in words_iot: if i.lower() in x.lower(): return 'Internet of Things' else: return np.nan

def fiveg(x): for i in words_5G: if i.lower() in x.lower(): return '5G' else: return np.nan

def metaverse(x): for i in words_vr_ar: if i.lower() in x.lower(): return 'AR_VR' else: return np.nan

def robotic(x): for i in words_robotics: if i.lower() in x.lower(): return 'Robotics' else: return np.nan

def policy(x): for i in words_regulation_policy: if i.lower() in x.lower(): return 'Reg_Policy' else: return np.nan

def literacy(x): for i in words_digitalliteracy: if i.lower() in x.lower(): return 'DHliteracy' else: return np.nan

data['AI'] = data['Title'].apply(ai)

data['IoT'] = data['Title'].apply(iot)

data['fiveg'] = data['Title'].apply(fiveg)

data['metaverse'] = data['Title'].apply(metaverse)

data['robotic'] = data['Title'].apply(robotic)

data['policy'] = data['Title'].apply(policy)

data['literacy'] = data['Title'].apply(literacy)

data.to_excel('Healthyageingdata_labeled.xlsx')
